# The Intraoperative Use of Defensive Antibacterial Coating (DAC^®^) in the Form of a Gel to Prevent Peri-Implant Infections in Orthopaedic Surgery: A Clinical Narrative Review

**DOI:** 10.3390/ma16155304

**Published:** 2023-07-28

**Authors:** Daniele Pressato, Angela Battista, Marco Govoni, Leonardo Vivarelli, Dante Dallari, Antonio Pellegrini

**Affiliations:** 1 Clinical and Scientific Affairs, Novagenit S.r.l., 38017 Mezzolombardo, Italy; 2 Quality Assurance and Regulatory Affairs, Novagenit S.r.l., 38017 Mezzolombardo, Italy; a.battista@novagenit.com; 3 Reconstructive Orthopaedic Surgery and Innovative Techniques—Musculoskeletal Tissue Bank, IRCCS Istituto Ortopedico Rizzoli, 40136 Bologna, Italy; leonardo.vivarelli@ior.it (L.V.); dante.dallari@ior.it (D.D.); 4 Reconstructive Surgery and Septic Complications Surgery Center, IRCCS Istituto Ortopedico Galeazzi, 20161 Milan, Italy; antonio.pellegrini@grupposandonato.it

**Keywords:** periprosthetic joint infection, prevention, hyaluronan, biomaterials, antibacterial coating

## Abstract

Periprosthetic joint infections (PJIs) in arthroplasty and osteosynthesis-associated infections (OAIs) in reconstructive surgery still represent a challenging complication in orthopaedics and traumatology causing a burden worsening the patient’s quality of life, for caregiver and treating physicians, and for healthcare systems. PJIs and OAIs are the result of bacterial adhesion over an implant surface with subsequent biofilm formation. Therefore, the clinical pathological outcome is a difficult-to-eradicate persistent infection. Strategies to treat PJIs and OAIs involve debridement, the replacement of internal fixators or articular prostheses, and intravenous antibiotics. However, long treatments and surgical revision cause discomfort for patients; hence, the prevention of PJIs and OAIs represents a higher priority than treatment. Local antibiotic treatments through coating-release systems are becoming a smart approach to prevent this complication. Hydrophilic coatings, loaded with antibiotics, simultaneously provide a barrier effect against bacterial adhesion and allow for the local delivery of an antibiotic. The intraoperative use of a hyaluronan (HY)-derivative coating in the form of a gel, loaded with antibiotics to prevent PJI, has recently raised interest in orthopaedics. Current evidence supports the use of this coating in the prophylaxis of PJI and IRIs in terms of clinical outcomes and infection reduction. Thus, the purpose of this narrative review is to assess the use of a commercially available HY derivative in the form of a gel, highlighting the characteristics of this biomaterial, which makes it attractive for the management of PJIs and IRIs in orthopaedics and traumatology.

## 1. Introduction

Although having a relatively low incidence, periprosthetic joint infections (PJIs) after hip and knee primary arthroplasty and osteosynthesis-associated infections (OAIs) consequent to the placement of a fixation device in traumatology may represent a serious clinical concern causing severe discomfort [1] for the patient and difficult clinical management in the post-operative course [2].

The incidence of either PJI after primary arthroplasty of the hip or knee is reported within the range of 0.5–3% [3,4] while the actual number might be higher, since the detection of infection is not always feasible [5,6], but incidences as high as 5% in certain patient populations have been recorded [7]. Patients with rheumatoid arthritis, skin ulceration, diabetes, systemic lupus, and/or poor nutrition showed a statistically significant increase in infection rates. There was also a trend towards infection in obese patients, those with recent urinary tract infections, and patients taking oral corticosteroids. Increased operative time was also found to be a risk factor [8,9]. Often the consequence of an infection after the placement of an arthroplasty implant results in prolonged hospitalisation and the long administration of antibiotics, and in case of failure, surgical revision becomes necessary, with removal of the prosthesis and placement of a new implant. Therefore, in this case, the burden of the incidence of re-infection increases dramatically, as demonstrated in a systematic review and a meta-analysis, which has reported re-infection rates after one-stage and two-stage septic revision of 13.1% and 10.4%, respectively [10,11].

In the most severe cases, prolonged infection can lead to limb amputation and even death of the patient [12]. In addition to the consequences for the patient, periprosthetic infections following arthroplasty and infections related to the placement of an osteosynthesis device in traumatology represent a significant economic burden for national health systems. The direct medical-related patient cost from the healthcare provider in high-income countries for two-stage septic revision, with re-revision, ranges from US$66,629 to US$81,938. This is about 2.5 times the cost of one- or two-stage septic revision without re-revision (range: US$24,027–US$38,109), which can be near double the cost of aseptic revision without re-revision (range: US$13,910–US$29,213). The major cost components were the perioperative cost (33%), prosthesis cost (28%), and hospital ward stay cost (22%) [13,14]. Similarly, bone and surgical site infections after osteosynthesis are particularly difficult to manage and represent a tremendous burden in fracture management. One of the most feared and challenging complications in the treatment of musculoskeletal trauma is infection after fracture fixation (IAFF) or OAI, which can delay healing and lead to reduced limb function, permanent functional loss, and life-threatening septic conditions or even amputation of the affected limb [15]. Furthermore, even with prevention measures for OAI and IAFF, there is significant morbidity in 1–30% of all orthopaedic trauma cases [16].

The incidence of infection after the internal fixation of closed fractures is reported to be 1–2%, but patients with open tibial fractures are at a higher risk of infection, with rates ranging from 6 to 33% [17]. A more recent paper reported that the incidence for deep surgical site infection after open reduction and internal fixation (ORIF) of closed tibia plateau fractures in a cohort of 676 adult patients was 2.5% [18]. Like PJI, the healthcare resource utilisation and treatment costs for fracture-related infections (FRIs) and IAFF represent an economic burden. A European health economic study states that healthcare costs were five times higher and total length of stay (LOS) and six times longer for open tibial shaft fractures in patients with deep infection vs. those with no infection [19]. Another EU study demonstrated that the average direct cost of treating a severe open tibial shaft fracture was estimated to be €49,817 but increasing to €81,155 when infection occurred. In patients treated within 7 days after their injury, infection increased the average direct cost and LOS by 124% and 135%, respectively [20]. The conclusions were that severe open tibial fractures covered with free flaps cause over a year of loss of work; in addition, infection increases the direct costs of treatment by over 60% and roughly doubles the LOS. Deep surgical site infection (DSSI) after open reduction and internal fixation (ORIF) may be increased in populations affected by comorbidities. Among these are a higher BMI, ASA ≥3, diabetes, alcohol abuse, open fracture, subluxation/dislocation, incision cleanness grade 2–4, high-energy injury mechanisms, chronic heart disease, a history of allergy, and the use of antibiotic prophylaxis [21]. Specifically, after open reduction and internal fixation of the tibial plateau fracture, significant risk factors for surgical site infection are an open fracture, the severity of trauma, skin conditions, compartment syndrome, chronic disease, immunocompromised patients, the operative time, smoking, obesity, and external fixation [22].

PJIs are frequently associated with antimicrobial-resistant pathogens, such as methicillin resistant Staphylococcus aureus (*S. aureus* MRSA), coagulase-negative *Staphylococci* spp., Gram-negative bacteria, and *Enterococcus* spp. In 15% of cases, the pathogen responsible for the infection is not identified, thus complicating the screening of an optimal antibiotic treatment [23].

The pathogens frequently involved in OAIs are *S. aureus* (60%), *Staphylococcus epidermidis*, multiple bacteria, and *Pseudomonas aeruginosa* [24].

### 1.1. Pathogenesis of PJI and OAI

The pathogenesis of infections associated with PJI and fracture-fixation devices are related to microorganisms growing in biofilms, which make these infections difficult to treat. The reduction of metabolic substances and/or waste product accumulation in biofilms causes bacteria to switch to a slow-growing (sessile) state. Therefore, systematically applied antibiotics mostly do not reach the necessary therapeutic levels, making them more resistant to most antimicrobial agents than their planktonic form [25,26].

Concerning the timing of PJI onset and their classification, recommendations and guidelines are reported within the “Proceedings of the International Consensus Meeting on Periprosthetic Joint Infections” [27] that took place in Philadelphia in 2013, in which the classification defined an early infection as one that occurs within 3 months of index surgery. Infections with onsets between 3 to 24 months are delayed infections, and those occurring >24 months after index arthroplasty were classified as late. These classification systems are useful because they provide a description of pathogenesis, with the theory being that early infections may be the result of seeding during surgery, whereas late infections are likely acquired via haematogenous spread.

Based on the literature, the classification changes for IAFF and OAIs in orthopaedic surgery; Willenegger and Roth [28] and Metsemakers et al. [16] classify IAFF according to the different times following the onset of patient symptoms into three groups: early (less than 2 weeks), delayed (2–10 weeks), and late onset (more than 10 weeks) infection.

Given the relevance of PJI, IAFF, and OAIs, the related significant pathological impact of the health conditions of patients and the need to develop strategies to reduce the incidence of infection and related costs have led to the development of different approaches and implementations of the treatment with the development of appropriate algorithms. The two International Consensus Meetings on PJI and orthopaedic-related infection that took place in 2013 and 2018, respectively, and the International Consensus Meeting (ICM) on musculoskeletal infection have unanimously set and delivered specific guidelines and strategies to implement practices against PJIs, OAIs, and biofilm formation to improve patient care [27,29,30].

### 1.2. Strategies to Prevent PJI and OAI

Strategies involve preoperative, intraoperative, or postoperative phases. Preoperative measures are extremely important as they are the first line of defence. The preoperative assessment allows for a key step to screen and diagnose underlying comorbidities and optimise modifiable risk factors before elective surgeries. Although demographic risk factors are largely non-modifiable, several modifiable risk factors for PJI and OAI had been identified. Those factors include rheumatologic disease, obesity, preoperative anaemia, diabetes mellitus, smoking, alcohol abuse, and a history of steroid administration, which are significant modifiable factors [31].

The operating room (OR), when it is very crowded, has been documented as a major source of an increased number of particles in the OR air. Therefore, limiting staff and movement in the OR reduces the number of airborne particles spreading from the skin of staff and the possible contamination by air entering from outside [32].

Several previous studies have demonstrated that laminar airflow (LAF) aeration systems are associated with reduced airborne microbial contamination and a decreased rate of surgical site infection (SSI) [33]. Nevertheless, other studies did not show that the decrease in SSI is cost-effective, making the use of LAF systems doubtful [34]. Prophylactic intraoperative wound irrigation is a key component of infection prevention. This practice aims to remove and dilute body fluids, microorganisms, and cellular debris and may have a direct antimicrobial effect when additive antiseptic agents are used [35]. Antibiotic prophylaxis during total joint arthroplasty (TJA) has been demonstrated to be an important step in the prevention of infections. Recommendations from the ICM suggest that the ideal start time is within 1 h pre-operatively, so that a bactericidal concentration is achieved by the time the surgical incision is completed [36]. There is also evidence that continuing antibiotic treatment beyond 24 h is not crucial and could lead to bacterial resistance [37].

The use of preoperative and perioperative antimicrobial prophylaxis is a standard procedure to inhibit peri-implant infections, and its effectiveness is related to the timing of administration. Nevertheless, standard systemic antibiotic prophylaxis showed conflicting results, particularly regarding the ideal timing and the most effective preoperative skin antisepsis. Hence, the optimal duration of systemic antibiotic treatment with surgical concepts of curing the wound and device-related orthopaedic infections is still unclear. Systemic antibiotics may have limited efficacy in decreasing the risk of infection associated with the use of foreign bodies, such as prostheses, osteosynthesis, and arthrodesis devices [38,39].

The concern is even more relevant in presence of a persistent PJI that is strictly related to the presence of a biofilm. Bacteria can colonise the surface of an implant, forming a biofilm of an extracellular polysaccharide matrix (glycocalyx) that protects the bacteria from the antimicrobial action of systemic antibiotics. Planktonic bacteria may be eliminated well through the conventional use of antibiotics; however, phenotypically different forms of bacteria embedded in biofilms are not. Infection will persist until the surgical removal of infected implants and dead tissue. Long-term antibiotics and wound debridement with implant retention are known to be ineffective and should be reserved for patients unfit for major surgery [40,41]. Therefore, as confirmed by several authors, the local delivery of antibiotics may be considered an attractive and effective method in the management of biofilm-related PJI, as they provide high concentrations of local antibiotics while simultaneously avoiding complications from systemic toxicity [42,43,44].

Similarly, in OAI, local prophylactic antibiotic therapy may represent an additional benefit to prevent osteosynthesis-associated infection after the administration of systemic antibiotics. This results in a 60% reduction in the risk of early wound infections. Furthermore, to improve the standard of care, some authors suggest increasing the effectiveness of antibiotics, particularly by assessing systems to deliver antibiotics at the tissue–implant interface [45]. Other investigators point out the need for larger comparative studies to improve the evidence; nevertheless, they concluded that the findings support the consideration of augmenting the antibiotic prophylaxis regimen to include locally delivered antibiotics, proving that patients with severe fractures could obtain the greatest benefit from the prevention of infections [46].

A recent systematic review of contaminated open fracture wounds showed a considerable risk reduction when additional local antibiotics were applied. The application of local antimicrobials was considered a complementary powerful adjunct in the standard treatment of FRI and could be considered, especially in cases with a remaining bony defect or “dead space”. Local antibiotics may be particularly effective in chronic/late-onset infections, where the perfusion of systemic antibiotics is meaningfully reduced due to chronic tissue scarring [47]. In conclusion, by reviewing several recent articles we can assume that surface coating of an orthopaedic implant with an antimicrobial or an antibiotic-loaded carrier could reduce bacterial attachment and biofilm formation. Therefore, a novel complementary therapeutic option can be considered that potentially prevents implant-associated infection.

### 1.3. Local Carriers and Coating Systems

Focusing on local carrier and coating systems, in recent years, investigators have had a great deal of interest in the development of hydrophilic hydrogels, based on biopolymers that may be used alone or as carriers in combination with antibiotics [48,49]. These hydrophilic coatings work by preventing bacterial adhesion on the surface of the implants, thus inhibiting the formation of biofilms. Particular interest has stimulated, both experimentally and clinically, the use of hydrophilic gel-coating based on hyaluronan (HY). The rationale of this approach is that HY coatings have been demonstrated to change the hydrophobicity of an implant that might be a suitable substrate for bacterial colonisation into a hydrophilic surface that has been demonstrated to diminish the chances of bacterial attachment, particularly in the early post-operative phase following surgery and therefore biofilm formation [50,51].

Furthermore, these hydrogel-based coatings when loaded with antibacterial agents also provide a physical barrier against bacterial adhesion, eluting, locally, a temporary high concentration of the antibiotic [48,52,53].

**Figure 1:**
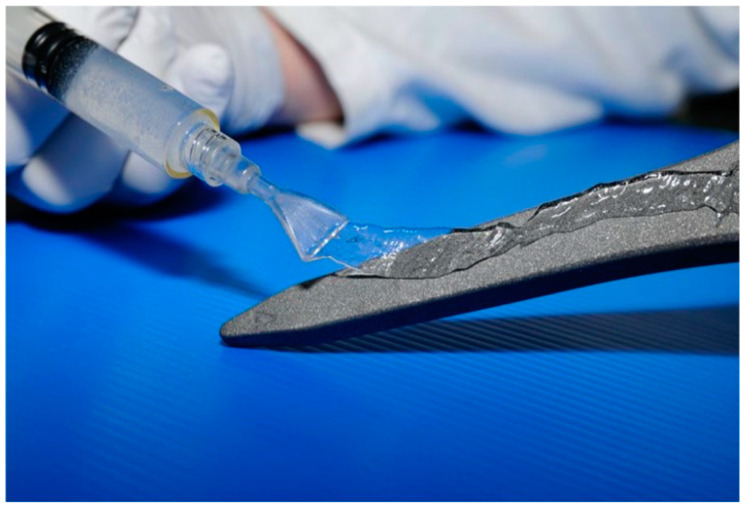
DAC^®^ gel spread uniformly over the surface of a prosthetic implant.

There are few coating systems currently available on the market. Among these, the Kit DAC^®^ (Defensive Antibacterial Coating—Novagenit S.r.l., Mezzolombardo, Italy), a biocompatible device composed of covalently bound HY and poly-D,L-lactic acid (PDLA), is able to provide a physical barrier against bacterial adhesion. The device is commercially available in the form of a powder, and when loaded with a sterile water solution of most common antibiotics in a range of concentrations between 2% and 5%, it forms a highly viscous gel that is spread over the implant surface (Figure 1).

The barrier action protects the implant surface against the adhesion and proliferation of bacteria as demonstrated by several in vitro [54], in vivo [55], and clinical investigations [56]. It subsequently undergoes complete reabsorption via hydrolytic degradation within 72 h, completely releasing the antibiotic.

Even with different rationales and indications compared to the DAC^®^ gel, only one other device is available on the market to prevent PJIs. Specifically, STIMULAN^®^ Rapid Cure (Biocomposites, Keele, UK) is an absorbable calcium sulphate antibiotic carrier in the form of beads designed to support the dead bone space and the local delivery of antibiotics to prevent deep site infection. Other calcium phosphate-based devices are commercially available for local antibiotic delivery, but the surgical indications of these compounds are different from the concept of the barrier action and antibiotic delivery, as performed by the DAC^®^ gel. These products act primarily against osteomyelitis or other bone infections that are difficult to heal. On the other hand, although there are products in the form of a gel with certain similarities to the DAC^®^ gel and based on biopolymers, such as chitosan and/or cerium oxide, they are currently in the preclinical in vitro [57,58] and in vivo (animal models) [59] development phase and are, therefore, not yet available on the market.

Therefore, focusing on the uniqueness of the DAC^®^ gel regarding its preventive “barrier” function against infections, the aim of this narrative review is to summarise the current relevant literature and evidence based on the intraoperative application of the DAC^®^ gel-antibiotic-loaded system, which makes its use attractive in orthopaedic surgery, including arthroplasty, traumatology, and vertebral surgery [54,60,61].

## 2. Materials and Methods

### 2.1. DAC^®^ Characteristics

From a chemical and molecular point of view, DAC^®^ is synthesised based on covalently bound HY and PDLA and is intended to undergo complete hydrolytic degradation within 72 h. The rationale for the use of HY and its derivatives has been previously demonstrated in vitro by several authors. Furthermore, data from the literature reported that HY-based coatings have been demonstrated to change the hydrophobicity of an implant surface into a hydrophilic surface, significantly reducing bacterial attachment [51]. Gasik et al. [50] described how a hydrophobic surface might be a suitable substrate for bacterial colonisation, while a hydrophilic surface can prevent biofilm formation, repelling bacterial adhesion over the implant surface. In addition, some authors have described that bio-matrices, such as HY, can provide an antagonistic effect against hyaluronidase, expressed by many pathogens to penetrate the physical defence of the host. Furthermore, the bacteriostatic effect of HY has also been demonstrated experimentally [62] and in different surgical applications, such as maxillo-facial surgery and dentistry surgery [63]. Based on the principles and rationale mentioned above, DAC^®^ hydrogel has therefore been selected as a hydrophilic physical barrier to avoid bacterial colonisation over the implant surface and consequently prevent biofilm formation. Moreover, the device showed synergistic activity when combined with various antibiotics and antibiofilm agents, reducing pathogen proliferation due to the controlled delivery of higher concentrations locally [54].

### 2.2. Study Selection and Data Extraction

We completed a literature search considering all papers relevant to the subject of DAC^®^ gel studies without time-limit filtering for clinical trials only. Articles not written in English or without full text available were excluded. The PubMed database was screened for any manuscript published at any time addressing the efficacy of DAC^®^ in the setting of biofilm-associated infections, DAC^®^ in the prevention of PJI, or DAC^®^ in the prevention of OAI. A PubMed-search was achieved using the following BOOLEAN operators: DAC AND (Defensive Antibacterial Coating) AND (Hyaluronan-PLA based Hydrogel) AND (Periprosthetic Joint Infection) AND (Implant Related Infection) AND (Osteosynthesis-associated Infection), in February 2023. Data were extracted from the full text using a piloted form that included the following study design: Randomised Controlled Trial (RCT), Case Report (CR), Prospective Case Series (PCS), Retrospective Case Series (RCS), Prospective Case Control Study (PCCS), Retrospective Case Control Study (RCCS), Retrospective Comparative Study (RCOS), Retrospective Cohort Analysis (RCA), and Retrospective Observational Study (ROS). Furthermore, the type of surgery, number of patients, follow-up, length, type of preoperative prophylaxis, type of postoperative prophylaxis, type of intervention, volume of DAC^®^ gel used, and type of control in RCT investigations were considered.

**Figure 2:**
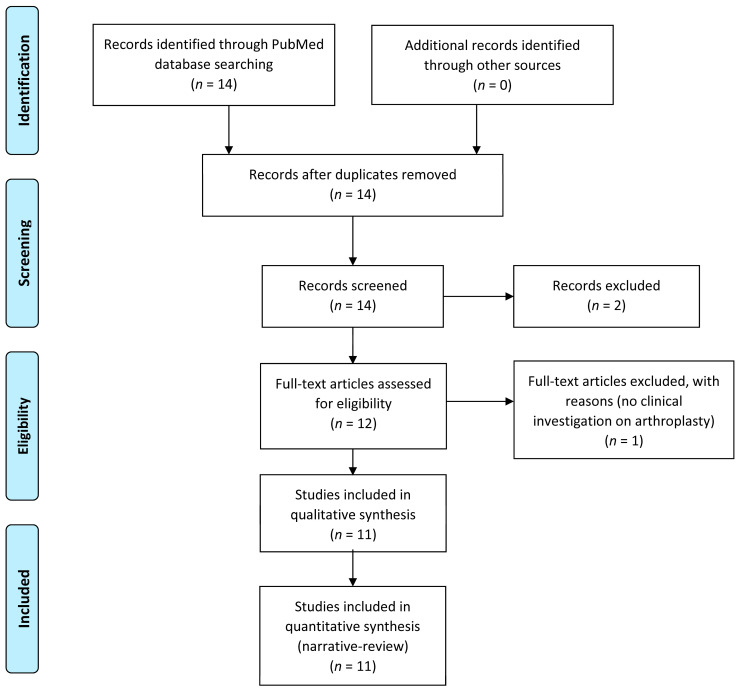
Flow chart of papers included in the review.

The flow chart of the selection procedure of the papers is represented in Figure 2.

## 3. Results

The research resulted in 14 publications issued between July 2016 and February 2023. However, 11 papers are included in this review because they match the search criteria, published in English and without duplicates or non-pertinent DAC^®^ use outside PJI and OAI prevention. There was a range of study designs, with two multi-centre RCT studies, three RCSs, two RCCSs, one multi-centre RCOS, one PCS, one multi-centre PCCS, and one ROS. Sample sizes belonging to the DAC^®^ treatment group in the included studies ranged from 10 to 189 cases, with an overall total of 572 patients (277 male, 295 female) and a mean length of follow-up range from 12.0 to 37.2 months. Three studies were carried out internationally and eight in Italy. The study and baseline characteristics of the participants are summarised in Table 1.

Romanò et al. [56] reported a significant reduction in the DAC^®^ antibiotic-loaded treated group in comparison to the standard control treatment. The rate of infection at a mean follow-up of 14.5 ± 5.5 months was 0.6% (*n* = 1) and 6% (*n* = 11), respectively (*p* = 0.003). Inclusion criteria were hip and arthroplasty, either primary or revision. In a further analysis, authors highlighted the fact that in the subpopulation undergoing a revision procedure, the infection rate dramatically increased, reporting a rate of infection of 13.4%, but no infection (0%) was observed in the DAC^®^-treated group. No allergic or other adverse events related to the DAC^®^ hydrogel coating were reported. Radiographic examination showed a lack of focal osteolysis around the implant in each group; nevertheless, progressive (>2 mm) radiolucent lines around the implant were observed in three patients in the treatment group and in seven patients in the control group. All implants were stable, and no signs of heterotopic ossifications were observed.

Malizos et al. [64] described the findings of a multi-centre RCT where the performance of antibiotic-loaded DAC^®^ to prevent surgical site infection in patients undergoing internal osteosynthesis for closed fractures was compared with the standard of care. Two hundred and fifty-six patients responding to the inclusion criteria were randomly assigned to receive antibiotic-loaded DAC^®^ or to a control group (without coating). At follow-up (18.1 ± 4.5 months), six surgical site infections (4.6%) were observed in the control group compared to none in the treated group (*p* = 0.03). No local or systemic side-effects related to the DAC^®^ hydrogel device were observed, and no detectable interference with bone healing was noted.

Capuano et al. [65] published a multi-centre case control study in which a cohort of patients affected by peri-prosthetic joint infection, undergoing one-stage procedure using DAC^®^ antibiotic-loaded hydrogel-coated implants, was compared with a retrospective series of matched patients treated with a two-stage procedure without the DAC^®^ hydrogel. The objective of the study was to demonstrate the hypothesis that a one-stage exchange procedure, performed with a DAC^®^ antibiotic-loaded hydrogel coating, provides a similar infection recurrence rate to a two-stage procedure without the coating in patients affected by peri-prosthetic joint infection. The primary endpoint was the rate of infection recurrence defined according to MSIS criteria [74]. Secondary endpoints were the length of the hospital stay after surgery, including the first and the second stage for two-stage procedures, the duration of antibiotic therapy, and the clinical scores at an average follow-up of 29.3 ± 5.0 months. Results showed the re-infection rate in two patients (9.1%) belonging to the control group and three patients (13.6%) in the two-stage group without statistically significant differences, demonstrating the effectiveness of using the DAC^®^ coating, with a reduced overall LOS and antibiotic treatment duration. The authors concluded that their findings warrant further studies on the possible applications of antibacterial-coating technologies to treat implant-related infections.

Zagra et al. [66] investigated the hypothesis that a two-stage exchange procedure, performed with antibiotic-loaded DAC^®^, could provide a superior reduction of the infection rate compared to a two-stage procedure without the coating, in patients affected by peri-prosthetic hip infection. Twenty-seven consecutive patients, undergoing a two-stage procedure, using cementless implants coated with the DAC^®^ antibiotic-loaded hydrogel, were compared with 27 matched controls, treated with a two-stage cementless revision procedure without the coating. DAC^®^ was used in combination with vancomycin (17 cases), teicoplanin, and ceftazidime in one case, a combination of vancomycin and rifampicin in one other case, and a combination of vancomycin and meropenem in seven patients. An average volume of 10.2 mL ± 1.3 mL of gel was used per patient. At a mean follow-up of 2.7 (range 2.1–3.5) years, no evidence of infection, implant loosening, or adverse events were observed in the DAC^®^-treated group, compared to four cases of infection recurrence in the control group. Furthermore, no significant differences were observed comparing the Harris Hip Score to evaluate the postoperative functional recovery, whereas the average total hospital stay, including rehabilitation, differed significantly between groups: 28.2 ± 3.9 and 33.8 ± 5.4 days in the DAC^®^ and in the control groups, respectively (*p* < 0.0001).

De Meo et al. [67] published data related to a retrospective observational investigation, in which they evaluated the onset of early postoperative infections in patients who underwent hip surgery with cementless prostheses treated with a DAC^®^ antibiotic-loaded hydrogel over their surface, in addition to systemic prophylaxis. Four patients were treated with DAC^®^ loaded with vancomycin + gentamicin, while 13 patients received DAC^®^ loaded with gentamicin only. The treated patients (*n* = 17) matched 1:1 with a control group of subjects that did not receive the hydrogel coating. The incidence of PJIs was assessed with a minimum of six months of follow-up, and the clinical outcomes showed no PJI onset in the DAC^®^ hydrogel-treated group versus six cases observed in the control group (*p* < 0.0001). No significant differences were reported regarding prosthetic osseointegration, functional results, and adverse events comparing the two groups.

Again, in in the same year, Franceschini et al. [68] described their experience relating to the surgical use of the DAC^®^ antibiotic-loaded hydrogel in a cohort of 28 patients who underwent elective uncemented two-stage revision total hip arthroplasty for chronic PJI. The type of antibiotic aqueous solution that DAC^®^ was hydrated with is not described in the paper, and the authors only reported the average volume (10 mL) of the device used. At an average follow-up time of 24 months (range 20–26), they found two early failures/re-infections after the two-stage protocol. Both occurred during the first 3 weeks after implantation, and patients underwent revision with implant removal and re-implantation during the same procedure. The remaining 26 patients did not show clinical or laboratory signs of re-infection after last the follow-up. In addition, investigators did not observe any loosening or failure of bone ingrowth of the implants.

A challenging demonstration of the effectiveness of the DAC^®^ antibiotic-loaded hydrogel was reported by Zoccali et al. [69]. In a case-controlled observational study, the authors assessed the usefulness of DAC^®^ in preventing PJI after the implantation of a mega-prosthesis implant for joint reconstruction with segmental resection for a bone tumour. This type of implant surgery is burdened by a relatively high complication rate when compared to primary joint replacements. The complications include infection, with an incidence following tumour resection ranging from 7.4% in metastatic tumours to more than 20% in sarcoma depending on the tumour location and associated co-morbidities [75,76]. Forty-three patients undergoing DAC^®^ coated mega-prosthesis were included in this investigation for oncological (*n* = 39) or non-oncological indications (*n* = 4) and were compared with a retrospective series of matched controls using a mega-prosthesis without a coating. At a mean follow-up of 2 years, no evidence of infection or adverse events was observed in the DAC^®^-treated group, compared to six cases of post-surgical infection in the control group (*p* = 0.028).

Corona et al. [70] retrospectively reviewed a cohort of patients (*n* = 10) having previously undergone multiple revision procedures with infected massive defects of the distal femur, treated with the Compress^®^ prosthesis in a two-stage procedure, together with a DAC^®^ antibiotic-loaded hydrogel. The specific protocol, microbiological data, clinical and radiological results, complications, functional results, and prosthesis survivorship were assessed. After a median follow-up of 27 months (range 12–50), no patient presented with recurrence of the infection, and limb salvage was achieved in all cases.

Pellegrini et al. [72] confirmed the effectiveness of the DAC^®^ antibiotic-loaded hydrogel in infected one-stage revision total hip arthroplasty (THA). Ten patients underwent cementless one-stage revision and were retrospectively evaluated using the following inclusion criteria: presence of a known organism with known sensitivity, non-immunocompromised patients with healthy soft tissues with minimal or moderate bone loss. Clinical assessment included recurrent infection, objective examination functional recovery (Harris Hip Score), VAS pain score, and radiograph examination. A pre-surgery microbiological evaluation showed the presence of the following pathogens: coagulase-negative *Staphylococci* (*n* = 5), methicillin-resistant *S. aureus* (*n* = 4), *S. aureus* (*n* = 1). At the time of surgery, all previously implanted prostheses and cement were removed, and meticulous surgical debridement was achieved. One-stage-exchange with cementless implants was performed whenever an infecting micro-organism and sensitivity were established before surgery. Intra-operative sampling was performed to confirm pre-operative isolated bacteria. At a mean follow-up of 37.2 (range 24–60) months, none of the 10 patients had clinical or radiographic signs of recurrent infection, and both functional and pain scores significantly improved. No adverse effect correlated to the use of the device were observed. The authors concluded that one-stage revision THA with DAC^®^ hydrogel-coated implants represents a safe and effective procedure, providing infection eradication and satisfying subjective functional outcomes in patients with infected hip arthroplasty.

Most of the papers available on the DAC^®^ antibacterial coating refer to its use in hip and knee arthroplasty procedures, as well as revision and traumatology. For the first time in 2021, Parbonetti et al. [71] described data related to the use of a DAC^®^ antibiotic-loaded system in instrumented vertebral surgery, where, as reported by recent literature, the incidence of peri-implant infections is significantly higher than surgical joint arthroplasty procedures [77]. The investigators retrospectively collected data related to a cohort of 73 consecutive patients who underwent primary or revision instrumented lumbar vertebral fusion for a degenerative spinal disorder with segmental instability. All received a DAC^®^ gentamicin-loaded hydrogel coating to cover all implant surfaces, including screws and rods, before wound closure; 10 mL of hydrogel loaded with 5% of gentamicin was used. All patients were followed-up at 3, 6, and 12 months for infection onset (primary end-point), and fusion was assessed using static and dynamic plain X-rays at the last follow-up. The postoperative evaluations showed no adverse events in the early postoperative period, and none of the patients in the cohort developed an early infection 2 weeks after surgery or at 3, 6, or 12 months of follow-up. After 12 months, radiographic examination showed that stabilisation and fusion were achieved in all the patients.

Recently, De Meo et al. [73] issued the results of an observational study confirming the efficacy, the safety, and clinical appropriateness of the use of a DAC^®^ antibiotic-loaded hydrogel in traumatology for the prevention of fracture-related infections (FRIs). A retrospective observational study was carried out on 37 patients with upper and lower limb fractures treated with internal fixation or prosthetic replacement, using a gentamicin-coated nail (CN) (*n* = 10) or antibiotic (gentamicin)-loaded DAC^®^ hydrogel (*n* = 27) applied to the implant of choice. Of the 37 patients examined, 14 (37.84%) were poly-trauma patients, while 13 patients had an open fracture. Clinical outcomes at the mean follow-up time (34.41 ± 9.46 months) with a minimum of 12 months showed that only one patient developed an FRI.

The summary of the treatment characteristic in each study and relative achievements are summarised in Table 2.

## 4. Discussion

Even though the issue of PJIs and the OAIs may be irrelevant, given their actual low incidence, it is important to consider that these pathological conditions are gradually increasing, because of the growth of arthroplasty and traumatology procedures closely related to the increase in the elderly population who is often affected by comorbidities [78,79]. Therefore, in recent years, this topic has triggered a heated debate among orthopaedic surgeons about identifying which therapeutic approach is the best one to adopt for the prevention of PJIs in joint arthroplasty and in and OAI traumatology. Recently, efforts were made to adopt a multidisciplinary approach to improve the diagnostic and treatment strategies related to PJIs and OAIs. There is increasing evidence that multidisciplinary approach and collaboration between healthcare workers are crucial to achieve these goals and to improve patient clinical outcome in the prevention of PJIs and OAIs [27,30,80]. For this reason, the focus of research and development has moved towards preventive rather than treatment strategies, as preventive strategies are considered more likely to have a greater overall impact on healthcare costs and patient outcomes. An example of an effective approach is the local delivery of antibacterial compounds from specific biomaterials formulated as coatings on devices [46,81,82]. Modification of the prosthesis surface to reduce bacterial colonisation and subsequent infection has also demonstrated promising clinical outcomes in the field of TJA and traumatology [83]. At present, a variety of surface modifications exist, such as employing antibiotics, silver [84], and copper (experimentally) [85], while in some cases, the occurrence of adverse events has raised some safety concerns [86]. Promising results in vitro, in vivo, and in clinical trials have been achieved using polymeric hydrophilic coatings loaded with antibiotics or inorganic absorbable beads [87,88]. Nevertheless, DAC^®^ is the only biological coating device based on biopolymers (hyaluronan) available on the market with the specific function of protecting the implant surfaces from bacterial adhesion and biofilm formation, and, at the same time, it is capable of allowing for the local release of antibiotics. In this regard, STIMULAN^®^, a calcium-phosphate medical device based on the use of silver coatings or washings with antiseptic solutions of the prosthetic surface, although having reached the market for clinical use, has different indications and chemical formulations compared to the DAC^®^ gel. Finally, although similar physical coatings based on biopolymers, such as chitosan and/or cerium oxide, are available, their used is still limited to preclinical evaluations.

Therefore, in this context, the present clinical narrative review has been prepared specifically to arouse the interest of the clinical–scientific and orthopaedic community in DAC^®^ gel antibiotic-loaded applications to effectively prevent the bacterial colonisation of an implant surface and avoid biofilm formation. The DAC^®^ antibiotic-loaded hydrogel represents a relevant clinical improvement in the prevention of PJIs end OAIs. Proven clinical efficacy has been demonstrated, particularly in those higher risk procedures, such as revision arthroplasty and mega-prosthesis implant surgery which, to date, still demonstrate a high rate of septic re-revision. Moreover, the device has demonstrated usefulness in reducing post-implant infection in orthopaedic procedures other than arthroplasty and traumatology. A relevant example is the application in vertebral surgery where the incidence of infection could be higher in relation to how many vertebral levels are affected by instrumented arthrodesis. The application of DAC^®^ and the local delivery of antibiotics within 72 h, the time within which the hydrogel is absorbed, avoid bacterial adhesion and the proliferation of pathogens with consequent biofilm formation. This is a crucial aspect, as preventing contamination in the initial phase of surgery means avoiding early and late implant/surgery-related infections that mainly occur within one year of surgery and finally improving osseointegration. All the prosthetic implants positioned and covered with the DAC^®^ antibiotic-loaded system showed complete osseointegration with the recipient bone; therefore, no implant mobilisation was detected.

Thus, the clinical investigations summarised in this narrative review represent an innovative approach in trauma and orthopaedic surgery; this meets the rationale and the requirements of the modification of an implant material’s surface, turning it from hydrophobic to hydrophilic for better repelling of bacteria and at the same time providing the local delivery of high concentrations of an antibiotic. The eleven original clinical studies discussed in this narrative review showed mostly promising results in different surgeries involving the musculoskeletal system. Material-associated infections are a feared complication in trauma and orthopaedic surgery and are likely to increase in number due to antimicrobial resistance and the growing number of implant operations, as well as the increased number of elderly patients frequently affected by comorbidities. Therefore, DAC^®^ hydrogel technology may represent a safe, easy, and effective tool to fight PJIs and OAIs in orthopaedic surgery, traumatology and vertebral surgery. Furthermore, the technique appears able to overcome many of the complications of treating PJIs, OAIs, and bone infections.

DAC^®^ antibiotic-loaded hydrogel scan be applied in a targeted way directly to the implant surface before wound closure, avoiding the need to administer additional systemic antibiotic treatment, preventing the risk of antibiotic resistance and other potential adverse events. DAC^®^ hydrogels, made of a combination of biocompatible and biodegradable polymers, locally release the antibiotics over a period of days, until the tiny gel layer dissolves without interference with bone healing and osseointegration.

Before concluding this narrative review, the authors believe it is also important to mention a relevant pharmaco-economics paper relating to the financial impact for the National Health Service following the application of the DAC^®^ gel in total joint arthroplasty procedures [89]. The analysis highlights the local protection of an articular implant through DAC^®^ gel application in a selected population, especially in subjects suffering from comorbidities with a high risk of contracting an infection, allowing for significant cost savings. Financial impacts include direct and indirect costs for managing infections, especially for the burdens associated with revision procedure costs, achieving economic balance already during the first year.

## 5. Conclusions and Future Perspectives

Surface modifications and the local delivery of antimicrobial substances complementary to systemic antibiotic therapy are promising and appear to be heading in a positive direction to reduce the risk of PJIs and OAIs, albeit further investigations are mandatory prior to consider novel medical devices as safe and effective.

Up to the present, the DAC^®^ gel was the only commercial product for PJI and OAI indications capable of effectively carrying and releasing antibiotics. Therefore, the authors believe it is appropriate to underline the uniqueness of the DAC^®^ gel with regards to surgical indications and its preventive “barrier” function against infections. Nevertheless, the authors recognise that this narrative review is limited to eleven clinical papers and that the majority is represented by case-control studies (level III evidence), with a shortage of prospective, and without randomised, investigations. Hence, further clinical trials with a higher level of evidence and designs involving subgroup analyses would be of interest to obtain data to further identify patients showing a higher risk of infection, who would be selected for treatment using this innovative technology for PJI and OAI prophylaxis. This aspect will be crucial to demonstrate the cost-effectiveness ratio of the treatment by assuring the spending decision-makers that the National Health Service can save costs by introducing the reimbursement of this treatment.

## 6. Patents

Antibacterial hydrogel and use thereof in orthopedics. WIPO Patent Application WO/2010/086421 A1.

## Figures and Tables

**Table 1:** Published clinical data of the DAC^®^ antibiotic-loaded hydrogel: study demography and baseline characteristics of the participants.

Authors	Year	Study Design	Type of Surgery	Sample Size		Mean Age		Average Follow–Up (Months)
				DAC^®^ gel + Ab	Non DAC^®^	DAC^®^ gel + Ab	Non DAC^®^	
Romanò et al. [56]	2016	RCT	Hip, knee arthroplasty—primary and revision	189	184	71 ± 10.6	69 ± 12.6	14.5 ± 5.5
Malizos et al. [64]	2017	RCT	Trauma	126	127	62.5 ± 21.2	58.6 ± 17.6	18.1 ± 4.5
Capuano et al. [65]	2018	PCCS	Septic hip arthroplasty revision (one-stage)	22	22	71.3 ± 13.6	71.9 ± 8.3	29.3 ± 5.0
Zagra et al. [66]	2019	RCCS	Septic hip arthroplasty revision (two-stage) uncemented	27	27	63.9 ± 11.7	64.8 ± 10.1	32.4 ± 7.2
De Meo et al. [67]	2020	RCCS	Aseptic hip arthroplasty revision	17	17	74.9 ± 11.5	75.9 ± 9.6	12.4 ± 5.7
Franceschini et al. [68]	2020	PCS	Septic hip arthroplasty revision (two-stage) uncemented	28	-	NR	-	24 ± 4.0
Zoccali et al. [69]	2021	RCOS	Arthroplasty, mega-prosthesis implant	43	43	45.6 ± 21.3	47.4 ± 19.5	24.2 ± 11.5
Corona et al. [70]	2021	RCS	Infected bone reconstruction	10	-	52.4 ± 11.1	-	27.0 (range 12–50)
Parbonetti et al. [71]	2021	RCS	Vertebral surgery, treatment of degenerative spinal disorders	73		61.6 ± 10.6	-	12
Pellegrini et al. [72]	2022	RCS	Septic hip arthroplasty revision (one-stage) uncemented	10	-	69.4 ± 8.3	-	37.2 (range 24–60)
De Meo et al. [73]	2023	ROS	Trauma	27	-	63.0 ± 24.84	-	34.41 ± 9.46

RCT, Randomised Controlled Trial; PCS, Prospective Case Series, RCS, Retrospective Case Series; PCCS, Prospective Case Control Study; RCCS, Retrospective Case Control Study; RCOS, Retrospective Comparative Study; ROS, Retrospective Observational Study; NR, Not Reported.

**Table 2:** Treatment characteristics in each study and relative achievements.

Authors	Year	Intraoperative Treatment with DAC^®^ Antibiotic-Loaded Gel and Related n. of Patients		DAC^®^ Volume (mL) Used Mean ± SD	Main Achievements
		Type of Antibiotic	n. of Patients		
Romanò et al. [56]	2016	DAC^®^ + vancomycin 5%	100	8.3 ± 2.7	Significant reduction in PJI in the DAC^®^ antibiotic-loaded group vs. control. Eleven early surgical site infections were observed in the control group and only one in the treatment group (6% vs. 0.6%; *p* = 0.003). No local or systemic side effects related to the DAC^®^ hydrogel coating were observed, and no detectable interference with implant osseointegration was observed.
		DAC^®^ + gentamicin 3.2%	70		
		DAC^®^ + vancomycin 2% + meropenem 2%	15		
		DAC^®^ + other associations	4		
Malizos et al. [64]	2017	DAC^®^ + gentamicin	78	5.7 ± 3.0	Significant reduction in PJI in the DAC^®^ antibiotic-loaded group vs. control. Six surgical site infections (4.6%) were observed in the control group compared to none in the treated group (*p* < 0.03). Wound healing, clinical scores, laboratory tests, and radiographic findings did not show any significant difference between the two groups. No local or systemic side-effects related to the DAC^®^ hydrogel product were observed, and no detectable interference with bone healing was noted.
		DAC^®^ + vancomycin	46		
		DAC^®^ + vancomycin + meropenem	2		
Capuano et al. [65]	2018	DAC^®^ + vancomycin 5%	14	10.2 ± 1.3	Two patients (9.1%) in the DAC^®^ group showed an infection recurrence, in comparison to three patients (13.6%) in the two-stage group. No significant differences were observed comparing the two groups. Clinical scores were similar between groups, while the average hospital stay and antibiotic treatment duration were significantly reduced after one-stage treatment with DAC^®^, compared to two-stage (18.9 ± 2.9 vs. 35.8 ± 3.4 and 23.5 ± 3.3 versus 53.7 ± 5.6 days, respectively).
		DAC^®^ + vancomycin + meropenem	8		
Zagra et al. [66]	2019	DAC^®^ + vancomycin 5%	17	10.2 ± 1.3	No evidence of infection, implant loosening, or adverse events was observed in the DAC^®^-treated group, compared to four cases of infection recurrence in the control group.
		DAC^®^ + teicoplanin 2.5% and ceftazidime 2.5%	1		
		DAC + vancomycin and rifampicin	1		
		DAC + vancomycin and meropenem	7		
De Meo et al. [67]	2020	DAC^®^ + vancomycin 2.5%	13	NR	No PJIs were reported in the DAC^®^-treated group, whereas six cases were observed in the matching control group (*p* < 0.0001). No significant differences were reported with regard to prosthetic osseointegration and functional results, nor were there side effects in the DAC^®^ treatment group.
		DAC^®^ + vancomycin 2.5% and gentamicin 2%	4		
Franceschini et al. [68]	2020	NR	28	10	Authors have found two early failures/re-infections after the 2-staged protocol. Both occurred during the first 3 weeks after implantation. Patients underwent revision with implant removal and re-implantation during the same procedure. The remaining 26 patients did not show clinical, laboratory signs of reinfection after the last follow-up. No loosening or failure of uncemented implants was recorded.
Zoccali C et al. [69]	2021	DAC^®^ + gentamicin 3%	23	9.4 ± 6.5	No evidence of infection or adverse events was observed in the DAC^®^-treated group, compared to six cases of post-surgical infection in the control group (*p* = 0.028).
		DAC^®^ + vancomycin 5%	9		
		DAC + other associations	11		
Corona et al. [70]	2021	DAC^®^ + vancomycin and gentamicin (concentration NR)	10	NR	Distal femur implants were used in a two-stage strategy, together with DAC^®^. At follow-up, limb salvage was achieved in all patients in this series of limb-threatening infected femoral injuries. No patient (10/10) presented signs of recurrence of the infection at the end of the follow-up.
Parbonetti et al. [71]	2021	DAC^®^ + gentamicin 5%	73	10.0	At 12 months of follow-up, no infection was recorded in the overall population. None of the patients reported significant pain or functional limitations after surgery. Post-surgically, computed tomography scans confirmed the correct positioning of the instruments.
Pellegrini et al. [72]	2022	DAC^®^ + vancomycin 5% and gentamicin 5%	10	5.0	None of the patients had clinical or radiographic symptoms of recurrent infection. A follow-up examination showed significant improvements in all variables [range of motion, Harris Hip Score (HHS), visual analogue scale (VAS) pain score] compared to pre-operative values (*p* < 0.05). Through radiographs, complete osseointegration of the implant was observed without progressive radiolucent lines or change in the position of the implant.
De Meo et al. [73]	2023	DAC^®^ + vancomycin 2.5%	27	7.5 ± 3.5	Only one case (2.94%) showed the onset of FRI at 5 months after surgery. Local antibiotic prophylaxis by coating resulted in a reduction in the incidence of FRI, as compared to the estimated preoperative risk. The use of the DAC^®^ antibiotic-loaded gel allows for the choice of antibiotic.

NR, Not Reported.

## Data Availability

Not applicable.
